# Ruts and Routine

**Published:** 1900-07-21

**Authors:** 


### RUTS AND ROUTINE.

Speaking at the London Polyclinic, Dr. William Osler, the well-known professor of medicine at the Johns Hopkins University, declaimed against the narrowness of much of our medical education, and the routine of much of our medical practice. A serious defect may, he says, warp the student's course from the very outset, for our students study far too much under one set of teachers. They do not move about enough. The student at a medical school develops a sense of local attachment to a place and to his teachers, which is very natural and very nice, but not always the best thing for him. "He goes out with a strong bias already in his mind, and is ready to cry 'I am of Guy's,' 'I am of Bart's,' 'I am an Edinburgh man!'" These local trammels badly handicap a man by giving him an arrogant sense of superiority which it is very difficult to escape from. "I knew three brothers in Canada," he says, "Edinburgh men, good fellows at heart, and good practitioners, but for them the science and art of medicine never extended beyond what their old teachers had taught. A Guy's man they could just endure, for the sake, as one of them said, of Bright, and Addison, and Cooper, but for men of other schools they entertained a supreme and really ludicrous contempt." How true this is! and how often we come across much the same lordly contempt of school for school even within the bounds of London. Next to the narrowness arising from school training comes that which results from falling into a routine in daily work. Into the clutches of the demon routine, says Dr. Osler, the majority of us ultimately come, for the mind, like the body, falls only too readily into the rut of oft-repeated experiences. Year by year we follow the same plan in practice, giving the same drugs, and settling down into routinists of a most commonplace type. He describes the mode of practice of a general practitioner as he saw it. "Kindly hopeful words, very sensible directions as to diet, and some half-dozen drugs seemed the essentials in his practice. . . . The dispensing, of the most primitive sort, was done at the table, on which stood four or five tin and paper boxes containing large quantities of calomel, soda, antipyrin, and Dover's powder. Other drugs, he said, were rarely necessary. He never used a stethoscope, he had no microscope or instruments of precision other than the thermometer. In reply to my questions he said that he rarely had to make an examination. 'If the patient has fever I send him to bed; if there is œdema I ask for the urine. Of course, I make many mistakes, and I sometimes get caught, but not oftener than the other fellows, and when I am in serious doubt I ask for a consultation.' This was a man of parts, a graduate from a good school; but early in his career he had become very busy, and, gaining the confidence of the people, and having much confidence in himself, he had unconsciously got into a rut." This, says Dr. Osler, is a by no means exaggerated picture of a routinist in general practice. But "we all have our therapeutic ruts, and we all know consultants from whom patients find it very difficult to escape without their favourite prescription, no matter what the malady may be." Men of this sort are stamped with the all-prevailing vice of intellectual idleness. It is so much easier to do a penny-in-the-slot sort of practice, in which each symptom is at once met by its appropriate drug, than to make a careful examination and really study the case systematically. Of all impediments to good work "ruts" are the most seductive and injurious.

